# Minimally Invasive Treatment of the Thoracic Spine Disease: Completely Percutaneous and Hybrid Approaches

**DOI:** 10.1155/2013/508920

**Published:** 2013-12-16

**Authors:** Tamburrelli Francesco Ciro, Scaramuzzo Laura, Genitiempo Maurizio, Proietti Luca

**Affiliations:** Department of Spinal Surgery, Catholic University of Rome, lg A. Gemelli 8, 00135 Rome, Italy

## Abstract

The aim of the study was to evaluate the feasibility of a limited invasive approach for the treatment of upper thoracic spine disease. Seven patients with type-A thoracic fractures and three with tumors underwent long thoracic stabilization through a minimally invasive approach. Four patients underwent a completely percutaneous approach while the other three underwent a modified hybrid technique, a combination of percutaneous and open approach. The hybrid constructs were realized using a percutaneous approach to the spine distally to the spinal lesion and by open approach proximally. In two patients, the stabilization was extended proximally up to the cervical spine. Clinical and radiographic assessment was performed during the first year after the operation at 3, 6, and 12 months. No technically related complications were seen. The postoperative recovery was rapid even in the tumor patients with neurologic impairment. Blood loss was irrelevant. At one-year follow-up there was no loosening or breakage of the screws or failure of the implants. When technically feasible a completely percutaneous approach has to be taken in consideration; otherwise, a combined open-percutaneous approach could be planned to minimize the invasivity of a completely open approach to the thoracic spine.

## 1. Introduction

Although widely used in the lumbar and lower thoracic spine, the minimally invasive approach is still limited in the treatment of the upper thoracic spine disease. The principal issue regarding treatment of thoracic spinal disorders through a minimally invasive approach is the potential for resulting neurologic injuries. The high risk of devastating complications is probably the primary roadblock to a wider use of the percutaneous approach to the thoracic spine.

Anatomic peculiarities and difficult clear visualization under fluoroscopy of the pedicles of the upper thoracic spine make percutaneous screw fixation a demanding procedure especially when multilevel thoracic fixation is required.

In order to limit the invasiveness of completely open surgery and to verify the feasibility of this demanding procedure, we began using a minimally invasive approach to the thoracic spine according to the following strategy. A completely percutaneous approach was carried out in well-selected cases in which the fluoroscopic visualization of the pedicles was optimal, while in cases where a completely percutaneous procedure was unfeasible, a combined, open-percutaneous approach to the thoracic spine was then performed. The surgical strategy we developed consists in percutaneous placement of pedicle screws in the lower and mid thoracic spine and conventional open pedicle screw insertion at the proximal portion of the fixation constructs.

We report our preliminary experience in treating thoracic spinal disorders requiring long stabilization. Four procedures were performed using a completely percutaneous technique and six through a combined, open-percutaneous approach.

## 2. Materials and Methods

**Figure 1:**
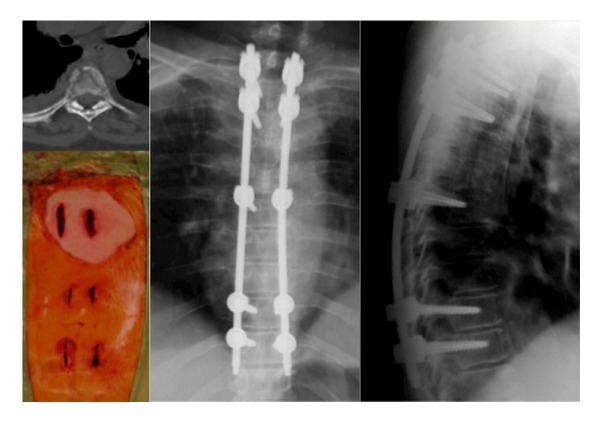
A 59-year-old female with fractures of T4 and T5, multiple, bilateral rib fractures, fractures of the spinous processes of T4/5/6/7, and lung contusion, treated surgically by posterior fixation with long instrumentation carried out percutaneously with screws implanted in T2-3/T6/T9-10.

**Figure 2:**
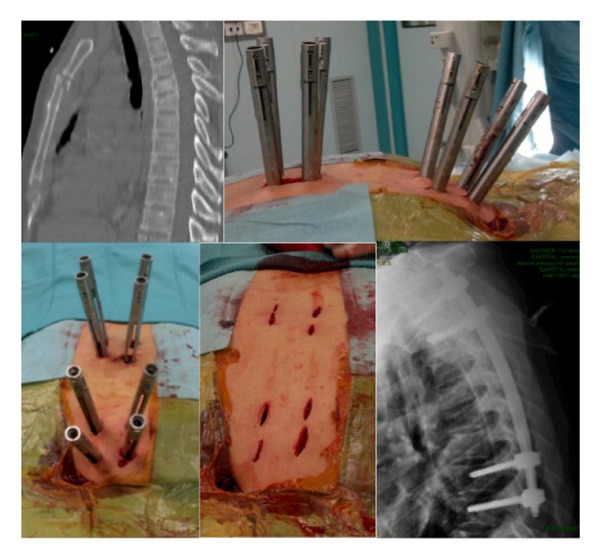
A 41-year-old man with traumatic fractures of T4-T5-T6 and fracture of the sternum. A completely percutaneous long fixation was carried out from T2-T3 till T8-T9. The patient was discharged from the hospital on the third postoperative day.

**Figure 3:**
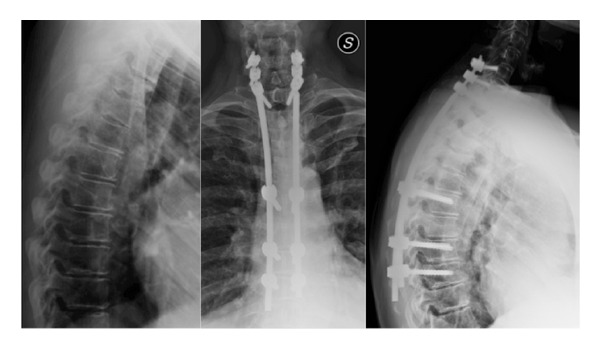
A 65-year-old female. Metastasis of breast cancer of T2-T3-T4. To limit the invasiveness of a completely open surgical approach, a hybrid long construct was carried out (percutaneous from T9-8 to T6 and open from T1, C7-6) using a double diameter rod.

**Figure 4:**
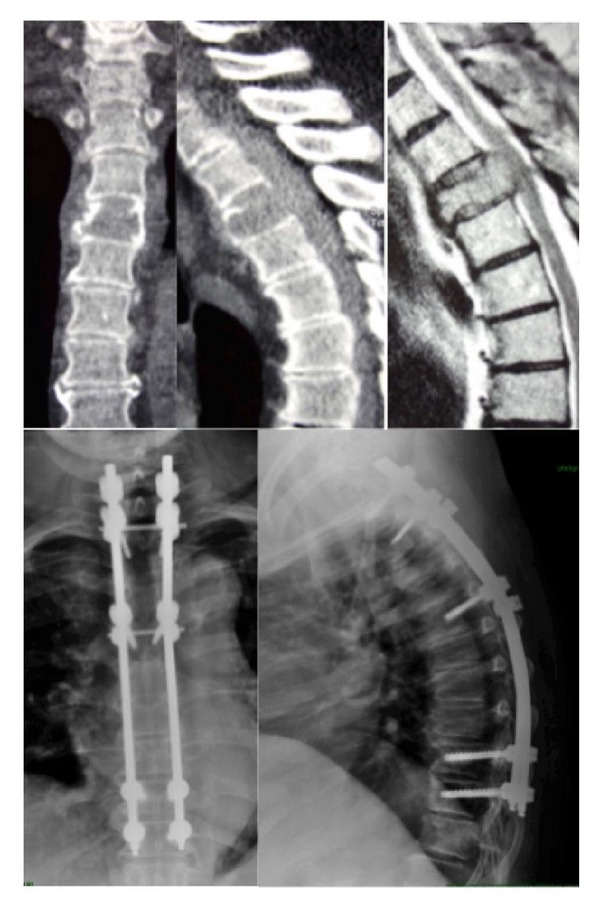
CT scan and MRI of a 71-year-old, obese female (120 Kg) with osteolysis of the T4. Hybrid construct performed percutaneously in the lower tract (T9-10) and by open approach in upper part (T1-2/5). No failure of the implant at 1-year follow-up X-rays.

We performed a long thoracic construct through an open-percutaneous combined approach (OPCA) in 6 patients (2 males, 4 females) and an entirely percutaneous approach in 4 patients (2 male, 2 female) (Table 1) using the minimally invasive PathFinder (Zimmer Spine) (Figure 1) and Viper system (DePuy Synthes Spine) (Figure 2). Seven patients (4 males and 3 females; minimum age, 41 years; maximum age, 71 years) had traumatic thoracic A3 fractures according to Magerl's classification, while the other three patients (all females) were affected by spinal tumors (Figures 3 and 4). T4 and T5 vertebral bodies were involved 5 and 7 times, respectively, T6 and T7 were involved in 2 cases. The most caudal pedicle screws were implanted in T12 while the most proximal were implanted in the lateral masses of C5. Associated bony lesions included multiple rib fractures, spinous processes fractures, fractures of the costotransverse joints, and bilateral fractures of the wrists in one case. The most severe associated lesions were lung contusion in three cases with slight impairment of respiratory function, traumatic pneumothorax in one case, and head injury in two cases.

**Figure 5:**
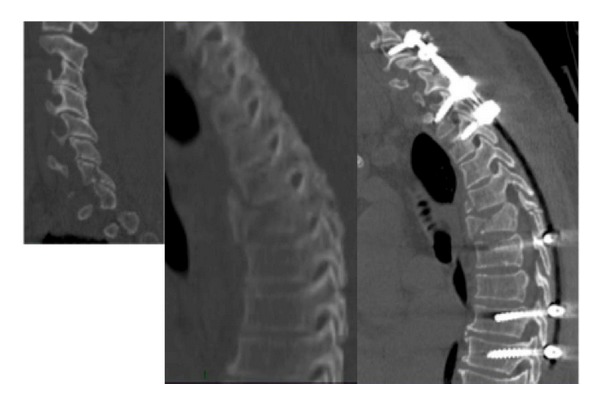
A 71-year-old man with traumatic fracture of the lower cervical spine and the T4-T5 vertebral bodies. A long fixation was carried out with hybrid procedure utilizing a double diameter rod. Pedicle screws of T6, T8, and T9 were implanted percutaneously.

Two of the OPCA constructs were extended proximally because of concomitant cervical lesions. One of these two patients had articular mass fractures of C6 and C7 with unilateral joint displacement requiring reduction and stabilization (Figure 5). Extension to the cervical spine in the other was necessary to ensure adequate anchorage proximal to neoplastic osteolysis of T2 (Figure 3). In both cases a specifically modified double diameters rod was used (Mountaineer, DePuy Synthes Spine).

In four cases (three fractures and one tumor) the implant was performed completely percutaneous. A very long construct was realized to obtain a more solid implant, able to better share loads of a long segment of the spine. The implant was extended from T2-3 proximally till T8-9-10 distally in case of fractures involving T4-5-6 vertebral bodies (Figures 1 and 2), while in the case affected by tumor, the neoplasia was localized at T6-7 vertebral bodies and the stabilization was carried out from T4 proximally to T12 distally.

One patient with tumor lesions had a long history of severe axial pain due to vertebral insufficiency before the onset of mild neurologic impairment (Figure 4). In this patient, who had multiple myeloma, computed tomography (CT) showed extensive destruction of the vertebral body and right pedicle of T4, while magnetic resonance imaging (MRI) showed tumor compression of the spinal cord at the same level. The other patient underwent surgery for breast cancer 5 years previously. During the last 2 months she experienced gradual onset of pain in the upper thoracic spine and minor neurologic impairment in the lower limbs. MRI showed neoplastic osteolysis of T2, T3, and T4 with compromise of the canal and mild cord compression. As a palliative procedure, we decided to decompress and stabilize the spine through an OPCA (Figure 3). Finally the third patient belonging to the tumoral group was admitted to our institution due to severe dorsal pain. She had history of breast cancer treated few years before. The preoperative MRI and CT scan demonstrated severe osteolysis of the T6 and T7 vertebral body. The fixation was carried out completely percutaneous except for a small incision focused on the lesion for taking samples for histological examination.

In 3 of 6 cases of OPCA the open exposure was extended distally to the level of the lesion either to allow autologous bone grafting in fractures or for spinal cord decompression in the tumor patients. Both of these procedures are unfeasible in a totally percutaneous way.

The surgical technique does not differ a lot from what we previously reported for long instrumentation of the thoraco-lumbar spine [1]. A crucial step at the beginning of the operation is the careful check of the optimal fluoroscopic view of the pedicles. The upper thoracic pedicles are generally better recognizable in anteroposterior (AP) views with the C-arm rotated in the craniocaudal plane according to the degree of kyphosis. Shoulders and soft tissues, especially in obese patients, frequently hinder visualization on lateral views. A second important technical feature is the right contouring of the rods before their implantation to preserve sagittal alignment, which varies substantially from patient to patient. This variability of thoracic kyphosis sometimes increased the difficulty of the procedure. Some patients had hyperkyphosis or worsening of preexisting pronounced kyphosis due to the vertebral lesion. Clinical assessment and implant surveillance were performed at 1, 3, 8, and 12 months. The accuracy of the pedicle screw placement was assessed at first postoperative control by means of CT scan with very thin slices in the three planes of the space and evaluated according to Youkilis's method [2]. Of 92 screws implanted, 84 were positioned inside the thoracic pedicles of which 22 by open and 62 by percutaneous approach. Eight screws were placed in the lateral masses of the cervical spine. The survival of the construct was assessed with standard X-rays at the subsequent controls till the first 1 y postoperative end-point, checking the failure of the implant, screw breakages or the presence of radiolucencies around the screws, signs of an impending loosening.

## 3. Results

No complications related to the surgical technique were observed and all patients showed satisfactory clinical outcome after a minimum 1-year follow-up. None of the patients had excessive intraoperative bleeding estimated maximum 250 cc and 100 cc for OPCA and completely percutaneous, respectively. Concerning the accuracy of the screws placement we observed that 22 of 24 open screws were good positioned and 2 were acceptable (more than 2 mm cortical violation) while 63 of 70 percutaneous screws were good positioned and 5 were acceptable. No infection or delayed wound healing was observed, including an obese patient (120 Kg), where the limited extension of the open approach facilitated mostly the postoperative nursing. No implant failed or loosened during follow-up. Patients treated for fractures started to walk in second day after the operation and were discharged from the hospital in 3–5 day postoperatively. Two patients belonging to the tumoral group were transferred to another hospital for postoperative rehabilitation because of the neurologic impairment. Both the neurologic patients had full recovery and all the patients because of the very limited approach were very quickly enrolled in the chemotherapy protocol treatment and oncologic clinical surveillance. At 1-year follow-up, they were still alive, able to walk without any external aids, and completely autonomous.

## 4. Discussion and Conclusions

Major thoracic spine fractures are generally caused by high-energy trauma, and, for this reasons, they are frequently associated with rib fractures and pulmonary contusions with severe impairment of respiratory function. The primary goal in treating patients with thoracic lesions is the rapid improvement of respiratory function to avoid sequelae and potentially fatal pulmonary complications. To obtain this goal, it is mandatory to stabilize the spinal injuries as soon as possible and in the less invasive way. The decision to stabilize with long instead of short constructs in the present series of patients was determined either by the association of multiple thoracic spinal fractures or the necessity of stabilizing vertebral structures compromised by cancer. When extensive instrumentation is required for proper support of thoracic vertebral lesions, there is necessarily a tradeoff between the desired mechanical efficacy and the debilitating procedures employed to obtain this efficacy. Until few years ago, it would have been unthinkable to use minimally invasive techniques to manage thoracic lesions because of the anatomical peculiarities of the region and the high risk of devastating complications [3]. With the advent of reliable percutaneous pedicle screw fixation systems, some surgeons recently tried to use this method in the treatment of thoracic fractures to minimize the invasiveness of an entirely open approach [4, 5]. Certainly the availability of the method relates to many aspects; the most important is the perfect visualization of the pedicle under fluoroscopy and its size and morphology. It is clear that the percutaneous approach allows consistent savings in terms of blood loss, recovery of the patient, and postoperative morbidity such as infection when compared to an open approach. Of course there are technical problems that need to be addressed, such as perfect visualization of the pedicle. Using fluoroscopically guided percutaneous insertion of thoracic pedicle screws, we relied more on AP views with the C-arm rotated in the sagittal plane according to the patient's kyphosis than on lateral views, where the shoulders, in the upper thoracic spine, tend to reduce detection of the vertebral bodies and pedicles. Perfect visualization of the pedicle, checked before starting the procedure, is essential for the insertion of the screw. On the AP view the pedicle appears as an oval within the limits of the vertebral body. This was the landmark we used for percutaneous pedicle screw placement. To ensure satisfactory purchase in the pedicle, we introduced the tip of the screw slightly medially without exceeding the medial border of the oval to avoid spinal canal encroachment and far from the superior edge of the vertebral body to avoid penetration of the disc space. The lateral view was limited to check the length of the screw. With this technique we did not experience major complications related to wrong screw placement except for a small incidence of uneventful violation of the cortical of the pedicle (2 for open and 4 for percutaneous). Park et al. using similar fluoroscopic guided technique reported very low screw malpositioning rates in their series of 172 screws postoperatively verified by CT [6]. If the tip of the screw remains lateral to the medial pedicle wall until the engagement of the vertebral body, it is highly improbable that it can cause a canal encroachment.

It may be argued that various navigation systems have been developed to reduce pedicle screws misplacement. Lieberman et al. reported that the use of robot guidance system increases the accuracy of percutaneous pedicle screw placement thereby reducing radiation exposure and procedure time [7], compared to the control group. They concluded that this advanced technology might also allow inclusion of patients with complicated anatomic deformities. Kakarla et al. treated percutaneously six patients affected by thoracic fractures (five acute unstable thoracic fracture and one osteoporotic burst fracture) with the assistance of intraoperative Iso-C C-arm fluoroscopy [8]. Accuracy of screw placement was investigated by postoperative CT scan according to the method of Youkilis et al. [2]. They concluded positively about the feasibility of percutaneous stabilization of complex spinal fracture with the aid of neuronavigation.

Undoubtedly the accuracy of the screw-navigated placement is higher in comparison with other “free-hand” techniques, but unfortunately the cost of navigation systems is often prohibitive. Even without a sophisticated pedicle screw-navigation system, neither our percutaneous technique nor our open “free-hand” technique was associated with intraoperative complications. Open “free-hand” pedicle screw insertion, guided by recognizable anatomical landmarks, is widely used at present, particularly for spinal deformities, even in the upper thoracic spine given the superiority of these constructs over hook constructs in terms of coronal and axial correction [9]. In the present series we experienced no difficulty using this technique in the upper thoracic spine, where percutaneous screw insertion would have been less feasible and more dangerous.

Although the limits of this work are the low number of patients and the short time of the outcome, we believe that they do not represent, however, a major limitation. In fact, the main purpose of our study was to verify the possibility to extend to the thoracic spine the advantages of minimally invasive procedure in the treatment of instability diseases in the same way we do in the lower spine. In this first phase of the study, we selected a limited number of patients that were eligible for less invasive approach with long stabilization. In particular, in the three cases of cancer it was important to assess the long-term outcome although it was mainly related to the underlying disease but the more important was to evaluate the possibility of achieving a good stabilization of the spine with fewer early and late complications. Given the impossibility to realize a completely percutaneous approach, an OPCA was carried out with the specific aim to reduce to the least the morbidity of the surgery in highly debilitated patients. Although palliative, the operation allowed a good general recovery and a strong support of the spine. Completely percutaneous approach in two cases of multiple fractures showed good clinical recovery of the patients with very fast discharge from the hospital 3 days after the operation. In two cases, we utilized an OPCA procedure limiting the proximal opening only to the segments chosen for pedicle screwing. In one case, the proximal approach was limited to the lower cervical spine and to the first two thoracic segments to allow the screwing of the cervical lateral masses and placement of the screws in the pedicles of T1 and T2. In the other case, the incision was prolonged to the fracture site to allow bone grafting. The need of posterior arthrodesis in the treatment of fractures is still a matter of discussion [10]. However, an OPCA can allow local bone grafting by distal extension of the proximal open approach. In conclusion, although a higher number of series with long-term outcome are needed to prove the reliability of the mininvasive techniques in the treatment of thoracic spine diseases, our series showed promising results at one-year follow-up with no complications technique-related.

## Figures and Tables

**Table 1:** Details of the patients. In the table are recorded the levels affected and the levels instrumented by completely percutaneous or combined (open-percutaneous) approach.

Pt no.	Sex/age	Fracture	Tumor	OPCA	Completely percutaneous	Other sites fractures	Comorbidities	Head injury	Neurologic deficit
1	M/41	T4, 5, 6	—	—	T2, 3/T8, 9	Sternum	Lung contusion	—	—
2	F/59	T4, 5	—	—	T2, 3/T6/T9, 10	Ribs, spinous apophysis	Lung contusion	—	—
3	M/54	T5, 6, 7	—	—	T3, 4/T8/T10, 11	—	—	Y	—
4	M/71	T4, 5	—	C5, 6/T1, 2 open T6/T8, 9 percutaneous	—	Wrists, C6, C7, ribs, spinous processes	Lung contusion	Y	—
5	M/65	T4, 5	—	T2, 3 open T6/T9, 10 percutaneous	—	Ribs	Pneumothorax	—	—
6	F/63	T4, 5	—	T2, 3 open T6/T8, 9 percutaneous	—	—	—	—	—
7	F/52	T5, 7	—	T3, 4 open T6/T8, 9 percutaneous	—	—	—	—	—
8	F/71	—	T4 myeloma	T1, 2/T5 open T9, 10 percutaneous	—	—	—	—	Y
9	F/65	—	T2, 3, 4 metastasis of breast cancer	C6, 7, T1 open T6/T8, 9 percutaneous	—	—	—	—	Y
10	F/57	—	T6, 7 metastasis of breast cancer	—	T4, 5/T11, 12	—	—	—	—

OPCA: open-percutaneous combined approach.
