# Erratum to “Minimally Invasive Treatment of the Thoracic Spine Disease: Completely Percutaneous and Hybrid Approaches”

**DOI:** 10.1155/2014/163231

**Published:** 2014-04-06

**Authors:** Francesco Ciro Tamburrelli, Laura Scaramuzzo, Maurizio Genitiempo, Luca Proietti

**Affiliations:** Department of Spinal Surgery, Catholic University of Rome, lg A. Gemelli 8, 00135 Rome, Italy

The authors' names were incorrectly listed as Tamburrelli Francesco Ciro, Scaramuzzo Laura, Genitiempo Maurizio, and Proietti Luca are corrected as stated above.

